# Schneider Membrane Elevation in Presence of Sinus Septa: Anatomic Features and Surgical Management

**DOI:** 10.1155/2012/261905

**Published:** 2012-07-17

**Authors:** Mario Beretta, Marco Cicciù, Ennio Bramanti, Carlo Maiorana

**Affiliations:** ^1^Implantology Department, School of Dentistry, University of Milan, IRCSS Cà Grande, MI, Italy; ^2^Human Pathology Department, Dental School, Messina University, Via Consolare Valeria 98100, Messina, Italy; ^3^Odontostomatology Department, School of Dentistry, University of Messina, Via Consolare Valeria 98100, Messina, Italy; ^4^Oral Surgery, Implantology Department, School of Dentistry, University of Milan, IRCSS Cà Grande, MI, Italy

## Abstract

Maxillary sinus floor elevation via a lateral approach is a predictable technique to increase bone volume of the edentulous posterior maxilla and consequently for dental implants placement. The sinus floor is elevated and it can be augmented with either autologous or xenogeneic bone grafts following an opening bone window created on the facial buccal wall. Maxillary septa are walls of cortical bone within the maxillary sinus. The septa shape has been described as an inverted gothic arch arising from the inferior or lateral walls of the sinus and may even divide the sinus into two or more cavities. Some authors have reported a higher prevalence of septa in atrophic edentulous areas than in nonatrophic ones. Radiographic identification of these structures is important in order to perform the right design of the lateral window during sinus lift. Aim of this investigation is to highlight the correct steps for doing sinus lift surgery in presence of those anatomic variations. Clinicians should always perform clinical and radiographic diagnosis in order to avoid complications related to the sinus lift surgery.

## 1. Introduction

The treatment of maxillary edentulous jaws with osseointegrated implants is often complex for the frequent pneumatisation of the maxillary sinus and for the remaining low-bone density and volume. The bone resorption, consequent to the loss of the dental elements, determines atrophy in height and thickness, by reducing the amount of available bone to the implant placement. In the 1970s, Tatum Jr [1] and then Boyne and James [2] developed the surgical technique of the maxillary sinus augmentation. The proposed approach represents the most reliable procedure for the bone reconstruction of the maxillary sinus. Sinus augmentation has evolved into a predictable surgical modality for increasing the existing height with bone of sufficient quality to allow successful placement of dental implants [3]. Sinus floor augmentation can be today considered a relative safe procedure, but severe complications may occur as a result of incorrect surgical plan or related to aggressive surgical manoeuvres [4]. Many different filling subantral materials have been used over the years [5]. Autologous bone represented for years the gold standard in bone grafting procedures for his osteoinductive, osteogenic, and osteoconductive abilities [6]. On the other hand, the pain deriving from the need of a double surgical site has prompted the researchers to develop alternative procedures using alloplastic, heterologous materials, and growth factors to support the bone regeneration [7–9].

Atrophy-related resorption of the alveolar process results in a vertical loss of bone volume, while progressive sinus pneumatization leads to an excavation of the alveolar process from the cranial aspect, which varies from one individual to another. Because atrophy-related resorption may occur differently in different areas of the alveolar process, bony septa can be considered residues between two such zones of resorption [10].

Knowledge of the maxilla anatomy, and moreover, of the blood supply of the maxillary sinus is mandatory to avoid unnecessary complications. [11–13].

All the surgical operations in the posterior maxillary region require detailed knowledge of maxillary sinus anatomy and possible anatomical variations. The aim of the present investigation is to underline how the presence of maxillary septa may influence the sinus floor augmentation surgical procedure. A complete knowledge of the patient's anatomical conditions is fundamental for exact planning of invasive surgery and helps to avoid complications.

## 2. Material and Methods

### 2.1. Literature Search and Selection

Several published paper underlined how dental implants positioned on posterior resorbed maxilla with extensive expanded sinus can be safely treated by a simultaneous sinus lift approach and implant insertion using the technical protocol and biomaterials studied with overall 10-years-long-term results [14, 15]. However, particular anatomical sinus features, like the presence of septa, can increase the percentage of complications of this safe technique.

The data from epidemiological studies on sinus septa prevalence on upper maxilla is not regular or predictable cause involving several additional topics. PUBMED research by “maxillary sinus septa” keywords evidences a total of sixty-one documents. However, only fifty-three manuscripts published and indexed in Medline assessing relation with “oral surgery diagnosis and therapy” and consequently published on related dentistry journal.

Fixot and Sorensons [16] dated on 1977 a document about retained root fragments along septa in the maxillary sinuses. Moreover, other fifteen manuscripts point out sinus septa prevalence, epidemiology, and anatomy.

A large number of studies (eighteen) involved radiological investigation on maxillary anatomy underlining how the volumetric analysis represents the more accurate way for performing sinus septa diagnosis. Nine published papers talk about sinus septa considering it on the sinus lift surgical procedure complications. Four animal studies and four cadaveric anatomy dissections and one systematic review complete the list of sinus-septa-related manuscript.

### 2.2. Data Collection

Referring about the full text data, the anatomical features and the surgical technique will be exposed thorough the paper in order to give clinicians complete information before performing sinus lift surgery.

In 1910, Underwood published a detailed description of maxillary sinus anatomy, evidencing antral septa of varying shape and size. Author divided sinus floor into three anatomic sections: a small anterior one over the premolar region a large median one descending between the roots of the first and second molars, and a small posterior one corresponding to the third molar region. These three sections of the floor of the sinus are usually underlined by ridges rising to distinct septa and connected to three defined periods of tooth activity, separated by intervals of growth time [17].

For decades, these septa were considered clinically insignificant anatomical variations. However, new diagnostic methods for verification of sinus disorders, such as endoscopy, have led to a different attitude towards the maxillary sinus and its anatomical variations [18, 19].

Krennmair et al. [20] divided septa into primary and secondary on another Septa classification: primary septa corresponding to those first described by Underwood, arising from the development of the maxilla and secondary septa arising from irregular pneumatisation of the sinus floor following tooth loss. Other authors [21–23] classified septa related to the presence/absence of maxillary teeth. Primary septa were located superior to a maxillary tooth; secondary septa were located on edentuolous maxillae. However a combination of both types has been recorded too. 

Furthermore, detailed knowledge of maxillary sinus anatomy has become increasingly important for sinus lift surgery [24].

**Figure 1:**
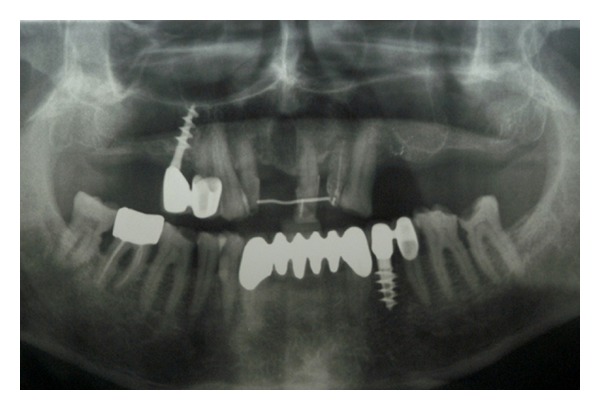
Panoramic rx shows the presence of possible septa in the left maxilla.

**Figure 2:**
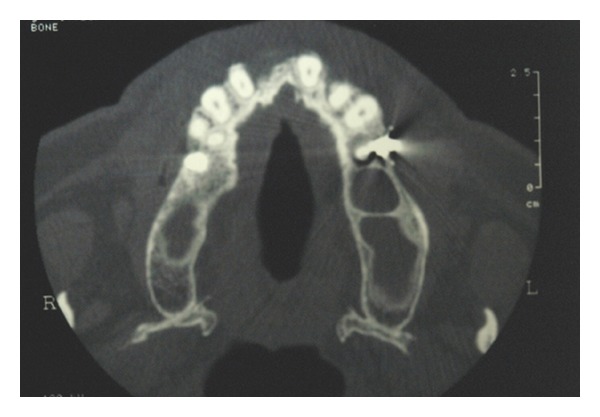
CT dental scan confirmed the presence of bone septa in the left maxilla.

**Figure 3:**
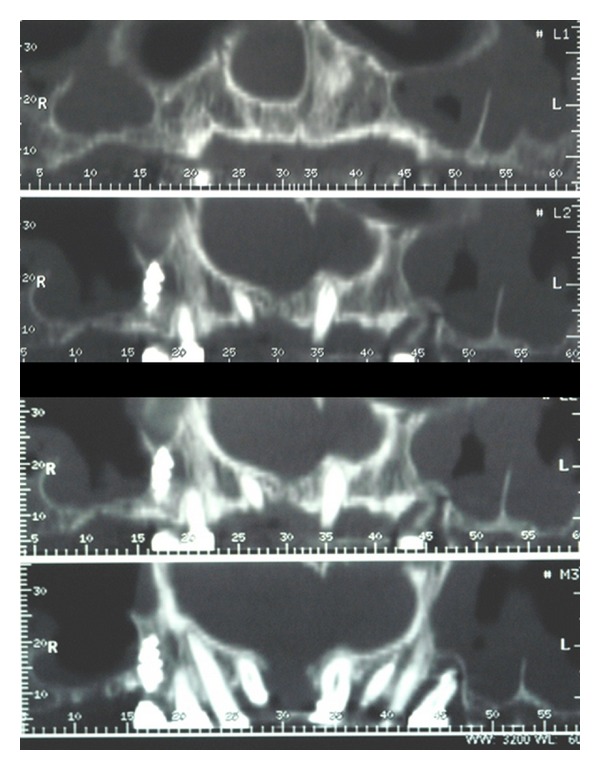
Axial view of the CT dental scan confirmed a deep septa in the left maxillary sinus.

**Figure 4:**
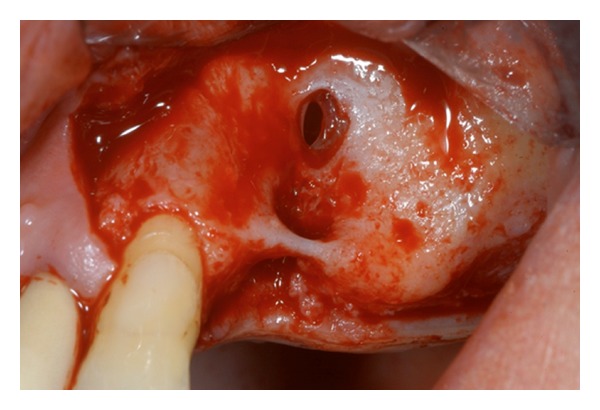
A mucoperiostal flap is elevated. The buccal wall shows residual ridge with a perforation of the Schneider membrane related to previous tooth infection and fistula.

The sinus lift technique, or internal maxillary sinus augmentation in the sense of sinus floor elevation, allows positioning of dental implants even when the posterior maxillary region has undergone severe bone resorption [25–27]. Before performing this kind of surgery, clinicians should suggest patients undergoing radiographic investigation for having a complete knowledge of the sinus extension [28–30]. Moreover, a CT dental scan of the upper jaw may give important information about the presence of septa and regarding the sinus three-dimensional limits (Figures 1, 2, 3 and 4).

**Figure 5:**
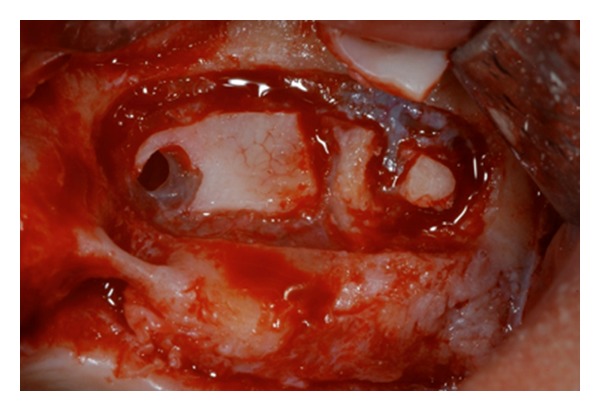
The buccal osteotomy is performed according to Boyne and James technique.

**Figure 6:**
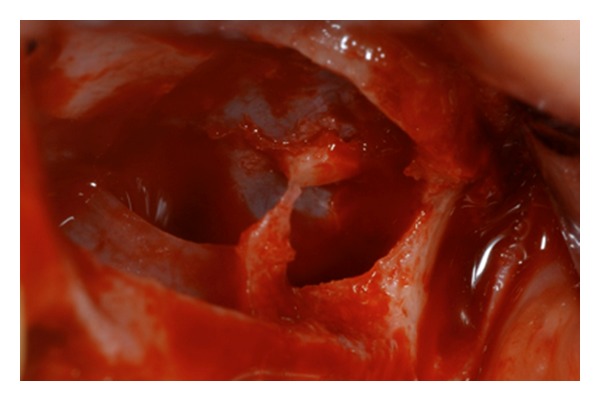
The presence of septa is well underlined after the sinus lift procedure performed. Two separate bone windows have been done.

**Figure 7:**
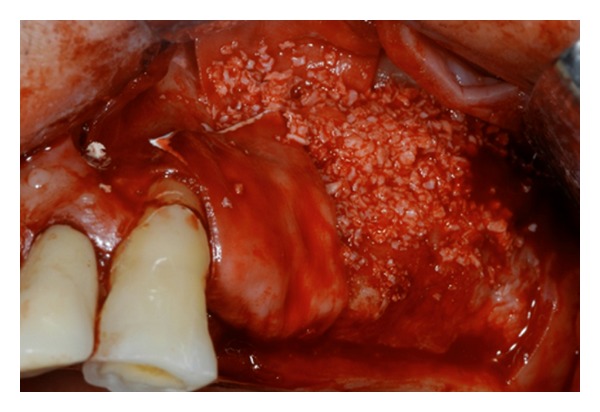
Deproteinized bovine bone has been used for covering the bone defect and for increasing the bone volume of the maxilla after the sinus lift.

**Figure 8:**
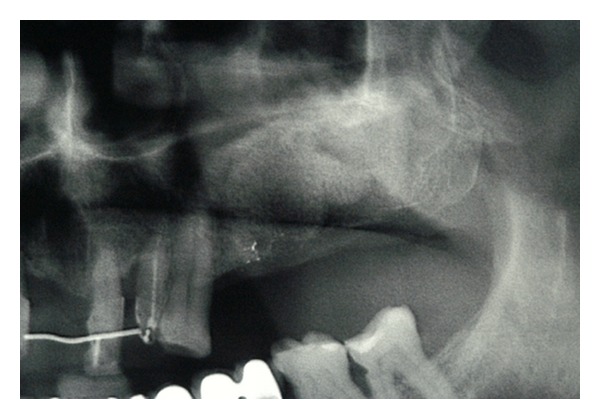
Panoramic rx control at 6 months after the surgery confirmed the newly bone formation.

In this surgical technique, a hinged window is made in the facial antral wall and inverted to create space for the grafting material. Either an autologous or a xenogenic bone graft is then placed between the former antral floor and the elevated sinus membrane, including or not inverted bone plate [31]. The presence of maxillary sinus septa can complicate both the luxation of the window into the sinus and the lifting of the membrane [20]. Boyne and James [2] advise cutting the septa with a chisel and removing them with haemostatic forceps, for placing the graft into the cavity without interruption. Sometimes, it is necessary to modify the buccal window design to avoid fracturing the septa: if the septa is high, it is advised to make two windows, one on each side [4, 32] or make one w-shaped window if the septa is lower [4] (Figures 5, 6, 7, and 8).

Although several modifications of this surgical technique have been proposed during the past few years, either with a supplementary or a simultaneous Le Fort I osteotomy, horseshoe osteotomy or nasal floor elevation [33], the original technique described by Boyne and James (1980) is still valid today [2].

**Figure 9:**
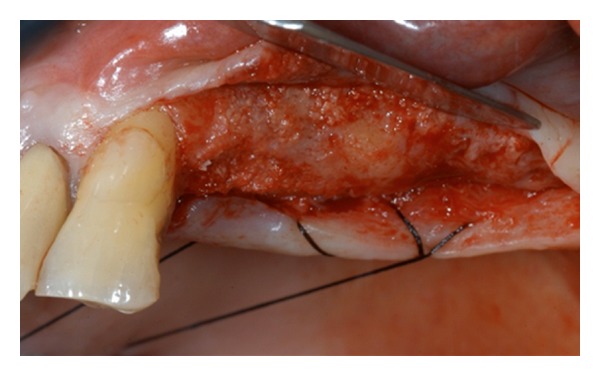
The new opened mucoperiostal flap clearly shows a good amount of bone formation.

**Figure 10:**
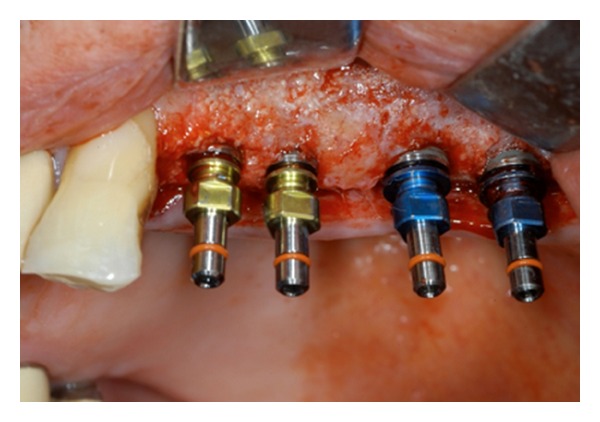
Four dental implants have been placed in order to perform prosthetic restoration of the previous edentulous area.

After a period of 6/9 months, dental implants can be positioned in the newly formed bone (Figures 9 and 10).

## 3. Discussion

The surgery procedures of the posterior maxillary region require detailed knowledge of maxillary sinus anatomy and possible anatomical variations. Detailed knowledge of the patient's morphological conditions allows exact planning of invasive surgery and helps to avoid complications. Several investigations analyzed the prevalence of sinus septa in the bone maxilla. Authors of those studies calculated the incidence number based on the number of sinus, which have septa, or on the number of subjects who have septa. The main results of those studies state how the antral septa are more commonly found in edentulous atrophic maxillae than in dentate maxillae. The septae in edentulous atrophic maxillae are usually shorter than those found in dentate maxillae. When present, maxillary sinus septae are more common anteriorly than posteriorly [23, 27, 30]. Additionally, the prevalence of septa has no relation with patient's sex or age, but there are variances based on the sorting of edentulism; some studies described a higher prevalence of septa in totally edentulous/atrophic areas than in partially edentulous/nonatrophic ones, with statistically significant differences [17, 21, 26]. Many authors contemplated the presence of septa if the height measured more than 2.5 mm [26, 30].

Despite the overall progress in dental implantology, dental implants positioning in the posterior atrophic maxilla are already considered to be a challenging procedure due to great levels of reduced bone volumes in many cases [34]. Grafting of the subantral space for augmentation is a prerequisite to overcome this deficiency [35]. Autogenous bone shows osteoinductive and osteoconductive properties and has, therefore, long been considered the material of choice for sinus augmentations. Because of its main disadvantages such as limited availability and donor site morbitdity various allografts, xenografts and alloplastic materials are used to substitute autogenous bone. Though bone graft materials give only few osteoinductive potential, they may act as a scaffold for bone growth [36]. In a recent review [37], the overall implant survival rate using 100% autogenous bone grafts for sinus augmentations was lower (88.9%) compared to combined grafts (94.7%) and 100% bone substitutes (96.1%). However, several studies (60%) associated with autogenous bone grafts referred the use of implants with machined surfaces that, added together, achieved poorer survival rates (86.3%) than textured surfaces (96.7%). The authors concluded that grafts of bone substitutes alone or in combination with autogenous bone were at last as effective as those exclusively constituted by particulate autogenous bone [36].

Sinus lift procedure performed by using xenograft materials is today a common and predictable technique. Histological and immunohistochemical investigations of human and animal biopsies taken after implantation of those bone graft showed signs of osteoconduction as well as osteoinduction, a high biocompatibility and a angiogenic response [37–42]. Autologous bone has been considered the gold standard for years, but its use could be limited by the donator's morbility, by its reduced availability, and by its variable resorption.

However, even if the surgeon may choose several kinds of materials for doing the graft, the problems related to the septa presence should be prevented and considered before doing the surgery.

Underwood observed the existence of another type of septa, indicating that it must have a different origin, as it seemed to be unrelated with teeth. Vinter et al. confirmed that resorption of maxillary alveolar process incomes irregularity in different regions, leaving bony crests on the sinus floor [3]. Consequently, incomplete septa on the sinus floor as known like “secondary septa” can be considered a result of tooth loss and bone resorption. Underwood was the first to study maxillary sinus septa and examined 45 dried skulls cut.

Ulm et al. [26] performed an observational study on the septa of 41 edentulous maxillae during sinus lift procedures underlining the anatomical features of the septa. Lugmayr et al. [23] observed the presence and morphology of maxillary sinus septa by observing the CTs of 100 adult patients. This investigation pointed out how the view of the maxilla can be useful for underlining septa presence. Krennmair et al. [20] in 1997 performed another analysis about 194 posterior maxillary regions, which were divided, into 4 group: Group 1 clinical observation during sinus lift procedure with panoramic Radiograph evaluation, Group 2 skull for anatomic evaluation, Group 3 TC evaluation of edentulous alveolar ridge, and Group 4 TC evaluation of dentate maxillary ridge [26]. The study showed the presence of different anatomies related to the patients age and teeth presence on the mouth.

According to several investigations, the diagnosis of the septa presence is fundamental in order to avoid surgical complications. The elevate number of false diagnosis established using panoramic investigation remarks how this kind of method cannot be suitable to entirely evaluate the sinus anatomic extensions. Otherwise, CT Scan, 3D, and Cone Beam investigation are today the better diagnostic investigation to underline the real maxillary anatomy highlighting the presence of septa.

## 4. Conclusion

The results of this study suggest how first-level radiographic investigation like orthopantomography or X ray are not appropriate for thorough evaluation of the sinus floor and its anatomical variants. Otherwise, CT and subsequent reconstructions consent high-resolution imaging of anatomical bone structures and can be considered the method of choice for imagining and investigating sinus septa presence. Specially, the CT axial section may help clinicians on evaluationg the septa orientation. Moreover, axial section is the ideal sectional plane to examine this bony structure.
